# Scalable Generation of Universal Platelets from Human Induced Pluripotent Stem Cells

**DOI:** 10.1016/j.stemcr.2014.09.010

**Published:** 2014-10-16

**Authors:** Qiang Feng, Namrata Shabrani, Jonathan N. Thon, Hongguang Huo, Austin Thiel, Kellie R. Machlus, Kyungho Kim, Julie Brooks, Feng Li, Chenmei Luo, Erin A. Kimbrel, Jiwu Wang, Kwang-Soo Kim, Joseph Italiano, Jaehyung Cho, Shi-Jiang Lu, Robert Lanza

**Affiliations:** 1 Advanced Cell Technology, Marlborough, MA 01752, USA; 2 Department of Pharmacology, University of Illinois College of Medicine, Chicago, IL 60612, USA; 3 Department of Medicine, Brigham and Women’s Hospital, Boston, MA 02115, USA; 4 Harvard Medical School, Boston, MA 02115; 5 Vascular Biology Program, Department of Surgery, Boston Children’s Hospital, Boston, MA 02115, USA; 6 Allele Biotechnology, San Diego, CA 92121, USA; 7 MacLean Hospital, Harvard Medical School, Belmont, MA 02478, USA

## Abstract

Human induced pluripotent stem cells (iPSCs) provide a potentially replenishable source for the production of transfusable platelets. Here, we describe a method to generate megakaryocytes (MKs) and functional platelets from iPSCs in a scalable manner under serum/feeder-free conditions. The method also permits the cryopreservation of MK progenitors, enabling a rapid “surge” capacity when large numbers of platelets are needed. Ultrastructural/morphological analyses show no major differences between iPSC platelets and human blood platelets. iPSC platelets form aggregates, lamellipodia, and filopodia after activation and circulate in macrophage-depleted animals and incorporate into developing mouse thrombi in a manner identical to human platelets. By knocking out the β2-microglobulin gene, we have generated platelets that are negative for the major histocompatibility antigens. The scalable generation of HLA-ABC-negative platelets from a renewable cell source represents an important step toward generating universal platelets for transfusion as well as a potential strategy for the management of platelet refractoriness.

## Highlights

• Large-scale production of platelets from human iPSCs under defined conditions
• iPSC platelets are functional both in vivo and in vitro
• Knockout of β2-microglobulin gene in iPSCs generates universal platelets

## Teaser

Lanza and colleagues report a method to generate functional platelets from human iPSCs in a scalable manner. iPSC platelets formed aggregates, lamellipodia, and filopodia after activation and circulated in animals and incorporated into developing thrombi in a manner identical to human platelets. HLA-ABC-negative platelets were also generated from iPSCs, which represents the first step toward generating universal platelets for clinical transfusion.

## Introduction

The vital processes of blood coagulation, clot formation, and hemostasis rely upon a sufficient supply of platelets within a person’s bloodstream. Transfusion remains the most effective way to increase a patient’s blood platelet count, yet limitations in the supply of platelets is a constant problem. A limited shelf-life (5 days) and the requirement for room-temperature storage increase the risk of bacterial contamination and pose the biggest challenge for maintaining ample supplies. In addition, patients who receive multiple platelet transfusions, such as those with various types of cancer, often develop platelet refractoriness due to HLA alloreactivity and subsequently require additional transfusions with HLA-matched donor platelets (Schiffer, 2001). Finding alternative sources of nonimmunogenic, high-quality platelets can help alleviate chronic shortages in the supply of platelets and reduce the risks for refractoriness.

Generating functional platelets in vitro has been the focus of many studies (Reems et al., 2010), yet many unresolved problems still exist. Human CD34^+^ cells from bone marrow (BM) and umbilical cord blood (CB) are capable of producing megakaryocytes (MKs) and platelets (Choi et al., 1995; Matsunaga et al., 2006), but production is donor dependent and the expansion capability of these cells is limited. Human embryonic stem cells (hESCs) and induced pluripotent stem cells (iPSCs) have also been used to derive both MKs and platelets using different methods (Lu et al., 2011; Pick et al., 2013; Takayama et al., 2008, 2010), all of which rely on mouse embryonic fibroblast (MEF) feeders and serum at some point during their culture. Since both MEF and serum can potentially be contaminated with xenogenic pathogens, their use increases the risk for an immunogenic reaction in humans.

“Feeder-free” substitutes for MEF, including Matrigel (BD), CELLstart (Life Technologies), recombinant proteins (Rodin et al., 2010), and synthetic polymers (Mei et al., 2010), can be used in the maintenance and propagation of human pluripotent stem cells (hPSCs). However, the propensity of feeder-free hPSCs to undergo massive cell death upon EB formation (Lu et al., 2013) (unpublished data) greatly reduces their differentiation potential into all cell lineages. Lastly, retroviral infection with three genes converted human dermal fibroblasts (Ono et al., 2012) cells to MK-like cells without reprogramming to a pluripotent state, and these MK-like cells were found to generate platelet-like particles only when injected into immunodeficient mice (Ono et al., 2012). The use of viral vectors and the inability to produce platelets from the MK-like cells in vitro limit the clinical applicability of this approach. Methods described above all fall short of providing scalable and/or fully functional platelets for clinical use. Considering that one transfusion unit contains approximately 300–600 × 10^9^ platelets, the efficiency of in vitro methods will need to be greatly improved if they are to be used clinically.

The objective of our current investigation is to develop a clinically adaptable method for large-scale production of iPSC-MKs and platelets under completely serum- and feeder-free conditions in vitro. Here, we describe a well-defined, three-step protocol to differentiate human iPSCs into MKs and functional platelets in less than 20 days. Importantly, this method does not utilize serum and animal feeders at any stage of differentiation, which greatly increases its ability to be developed for clinical use. Using this method, large quantities of cryopreservable MK progenitors were produced from human iPSCs, thereby allowing billions of platelets to be produced rapidly upon thawing of these cells. The improved scale of platelet generation provided enough material to perform light transmission aggregometry (LTA) of fully in vitro-generated platelets, as well as other important functional assays including platelet circulation kinetics in living animals. In addition, by knocking out the β2 microglobulin gene in iPSCs, we generated HLA major (A,B,C)-negative MKs and platelets. This can facilitate future clinical development of a renewable “universal” platelet source to help reduce the incidence of refractoriness in vulnerable patient populations.

## Results

### Efficient Serum- and Feeder-free Direct Differentiation of Human iPSCs to CD31^+^ Hemogenic Endothelial-like Cells and MK Progenitors

**Figure 1:**
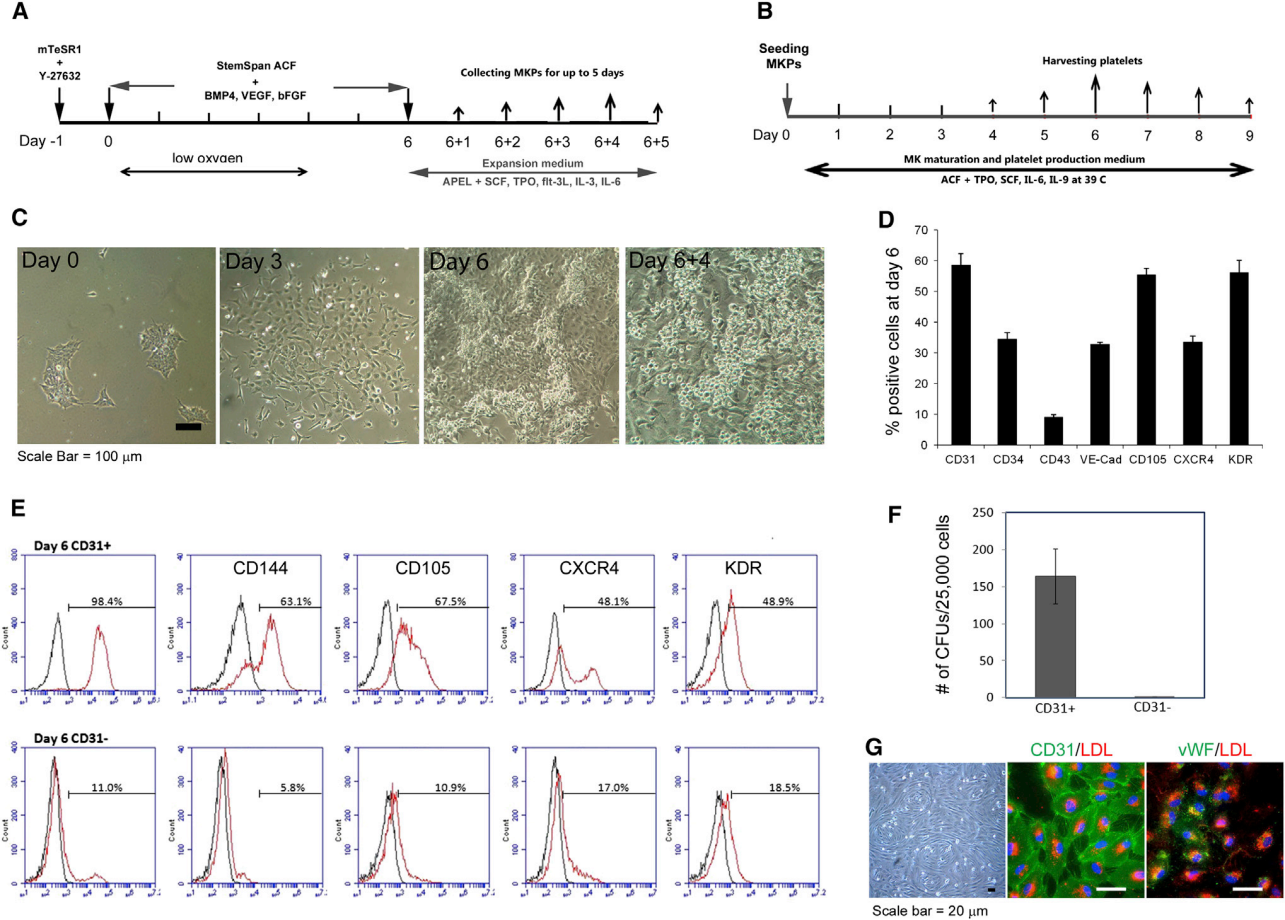
Generation of Megakaryocyte Progenitors from Human iPSCs (A) Schematic illustration of step-wise differentiation from iPSCs to MKPs. (B) Schematic illustration of iPSC MK maturation and platelet production. (C) Representative cell morphology at various stages of MK lineage specific differentiation (scale bar, 100 μm). (D) Cell-surface marker analyses of day 6 differentiation culture (mean ± SD, n = 3). (E) Representative flow cytometry profiles of cell-surface markers in day 6 CD31^+^ (upper panel) and CD31^−^ (lower panel) cells (black line, isotype antibody control; red line, specific antibody). (F) Colony-forming capability of day6 CD31^+^ and CD31^−^ cells (mean ± SD, n = 3). (G) From left to right: Morphology of endothelial cells derived from day 6 CD31^+^ cells; uptake of LDL (red) and staining of CD31 (green); uptake of LDL (red) and vWF staining (green) (scale bar, 20 μm).

Feeder-free iPSCs were differentiated into hemogenic endothelial-like cells and hematopoietic progenitor intermediates prior to further differentiation into MKs and platelets (Figures 1A and 1B). In the first 6 days of differentiation, we observed dramatic morphological changes: compact colonies (day 0) stretched out and became loose colonies (day 3), which grew out and formed sheets with a complex morphology by day 6 (Figure 1C). Cell-surface marker analysis on day 6 showed that 58.5% (±3.74%) of attached cells were CD31^+^. Approximately 34.43% of these cells were CD34^+^ and less than 10% of them were CD43^+^, indicating an early stage of hematopoietic commitment (Vodyanik et al., 2006). Further analyses demonstrated that between 30% and 60% of the attached cells also expressed CD144 (vascular-endothelial [VE] cadherin), CD105, CXCR4, and KDR (Figure 1D). As CD31 (PECAM-1) has been shown to be an important marker for hemogenic endothelium (Oberlin et al., 2002), we further determined the expression of several surface markers that are also expressed on hemogenic endothelium on both our CD31^+^ and CD31^−^ cells. Flow cytometry analyses demonstrated that about 67.5% and 63.1% of CD31^+^ cells expressed CD105 and VE cadherin, respectively, whereas only about 10.9% and 5.8% of CD31^−^ cells were positive for CD105 and VE cadherin (Figure 1E). Hematopoietic colony-forming assays showed that CD31^+^ cells possess robust hematopoietic potential with the development of colony-forming unit (CFU)-erythroid, CFU-myeloid, and CFU-mixture, whereas CD31^−^ cells developed very limited numbers of hematopoietic colonies (Figure 1F; Figure S1 available online). After plating on a fibronectin-coated surface, CD31^+^ cells formed a monolayer with characteristics of endothelium morphology, expressed high levels of von Willebrand factor (vWF), and took up acetylated low-density lipoprotein (LDL) (Figure 1G). These results demonstrate that CD31^+^ hemogenic endothelial-like cells can be efficiently generated by direct differentiation of feeder-free iPSCs under defined conditions.

**Figure 2:**
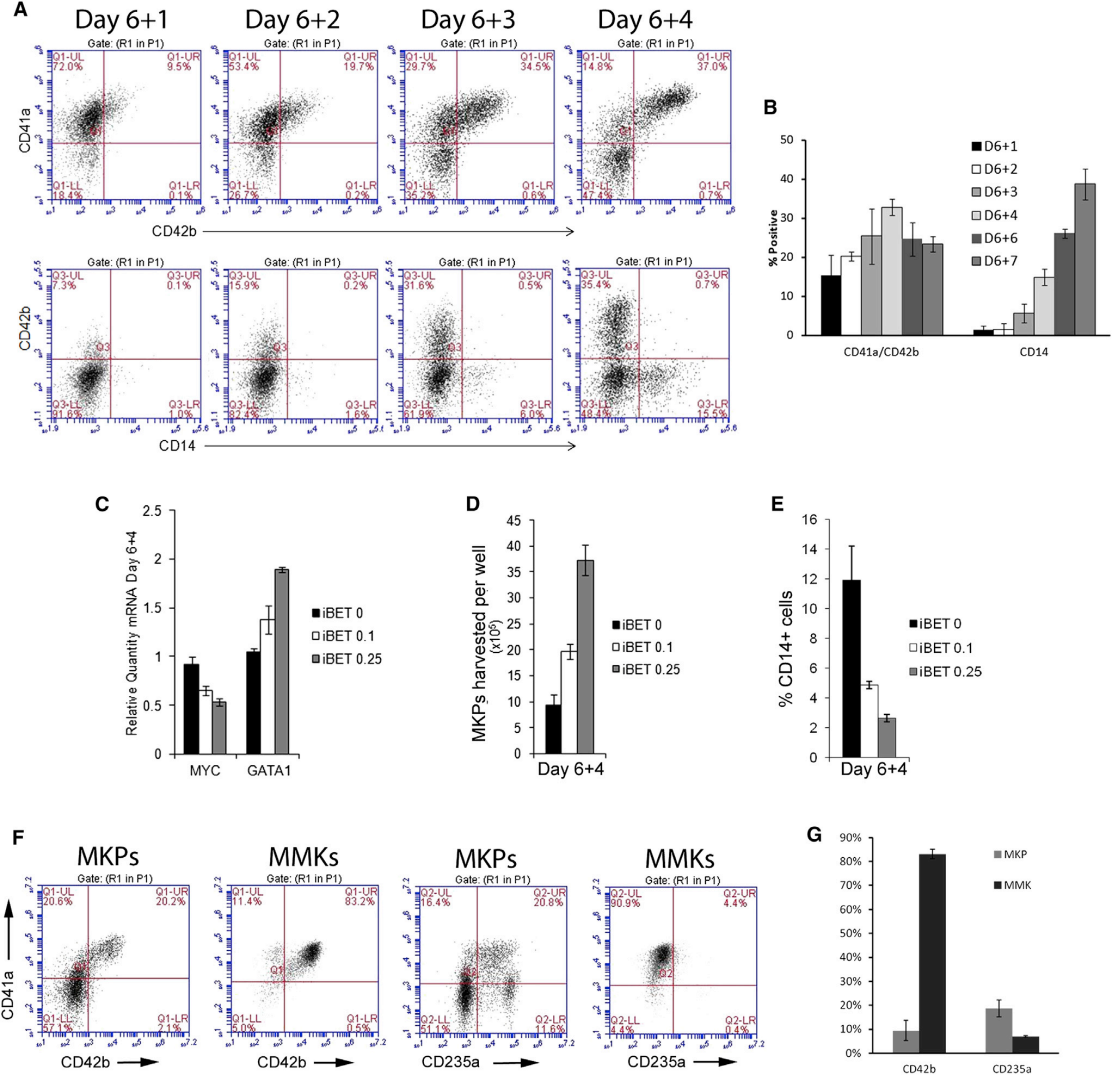
Effect of the myc-Inhibitor iBET on Differentiation of MKPs to Mature MKs (A) Representative results showing percentage of CD41a^+^CD42b^+^ and CD14^+^ MKPs from day 6 + 1 to day 6 + 4. (B) Time-dependent change of CD41a^+^CD42b^+^ and CD14^+^ MKPs percentage from day 6 + 1 to day 6 + 7 (mean ± SD, n = 3). (C) Dose-dependent effect of iBET on *MYC* and *GATA1* mRNA expression in MKPs (mean ± SD, n = 3). (D) Dose-dependent effect of iBET on MKP yield (mean ± SD, n = 3). (E) Dose-dependent inhibitory effect of iBET on CD14^+^ cells in MKP culture (mean ± SD, n = 3). (F) Representative results of CD42b and CD235a expression in MKPs and mature MKs (MMK). (G) Comparative expression of CD42b and CD235a in MKPs and MMKs (mean ± SD, n = 3).

Six days after initial differentiation, culture medium was replaced by expansion medium EXP-M. From day 6 + 1 to day 6 + 4, large numbers of rounded cells grew out of the attached cells as shown in Figure 1C. The majority of these floating cells were CD31^+^CD34^+^CD43^+^, indicating their commitment toward the hematopoietic lineage (Vodyanik et al., 2006). These floating cells were also CD41a^+^CD13^+^CD14^−^, and expression of the MK-specific marker CD42b was variable (9.5%–37%). We therefore defined these cells as megakaryocyte progenitors (MKPs) by cell-surface marker expression (CD31^+^CD34^+^CD43^+^CD41a^+^CD13^+^CD14^−^CD42b^+/−^) (Figure S2). However, we also observed that CD14^+^ myeloid cells (which are almost 100% CD42b^−^) increased from <1% to about 38% from day 6 + 1 to day 6 + 7, indicating the expansion rate of myeloid cells surpassed that of MKPs during this time period (Figures 2A and 2B). In five large-scale experiments, we generated a total of 2.06 × 10^9^ MKPs from 1.26 × 10^8^ iPSCs, an average of >16 MKPs per single iPSC input (Table S1).

### Downregulation of *myc* Promotes MKP Formation and Prohibits Myeloid Cell Expansion

Previous studies suggest that the c-*myc* gene may be involved in megakaryopoiesis (Chanprasert et al., 2006; Guo et al., 2009; Thompson et al., 1996). We therefore determined whether suppression of c-*myc* expression would enhance MK lineage differentiation/expansion and simultaneously prohibit myeloid and other lineage differentiation/expansion. MKP day 6 + 3 cultures were treated with noncytotoxic doses of a c-*myc* inhibitor, iBET151 (0.1 and 0.25 μM), for 24 hr and then harvested for analysis. Quantitative PCR analyses showed a dose-dependent downregulation of *myc* and upregulation of *gata1* in MKPs treated with iBET151 (Figure 2C). Supplementation of iBET151 at 0.1 and 0.25 μM resulted in ∼2- and 4-fold increases in MKP outputs, respectively, as compared to untreated controls (Figure 2D). Results also showed that addition of iBET151 from day 6 + 3 to day 6 + 4 inhibited the formation of CD14^+^ myeloid cells in a dose-dependent manner (Figure 2E). CD14^+^ myeloid cells were decreased from about 12% in control cultures to less than 5% and 3% in cultures treated with iBET151 at 0.1 and 0.25 μM, respectively. These results suggest that the c-*myc* gene not only plays a key role in early megakaryopoiesis, which is consistent with previous reports (Takayama et al., 2010), but also may be involved in the differentiation and expansion of other hematopoietic lineages.

### Feeder-free Generation of Platelets from iPSC MKs

We previously found that 40%–50% CD41a^+^/CD42b^+^ double-positive platelets were generated from MKs with stroma/feeder coculture whereas this amount decreased to <15% when feeder-free conditions were used (Lu et al., 2011). To overcome the requirement for feeders and improve production of CD41a/CD42b double-positive MKs and platelets, we tested several conditions with different basal media and cytokine/growth factor combinations. Our results showed that a combination of STEMspan-ACF medium with STEMspan Megakaryocyte Expansion Supplement (STEMCELL Technologies) as the core components of MK maturation medium was the best medium and cytokine mixture tested for iPSC MKP maturation and subsequent platelet production. As shown in Figures 2F and 2G, culturing of MKPs in this medium for 4 days resulted in a rapid increase in the CD41a^+^CD42b^+^ double-positive matured MK population from ∼20% to >80% and the almost complete elimination of CD235A^+^ erythroid cells. These results indicate that this MK maturation medium efficiently steers cells toward MK lineage development while suppressing the expansion of erythroid cells even though these cell types share a common bipotential progenitor (Klimchenko et al., 2009).

**Figure 3:**
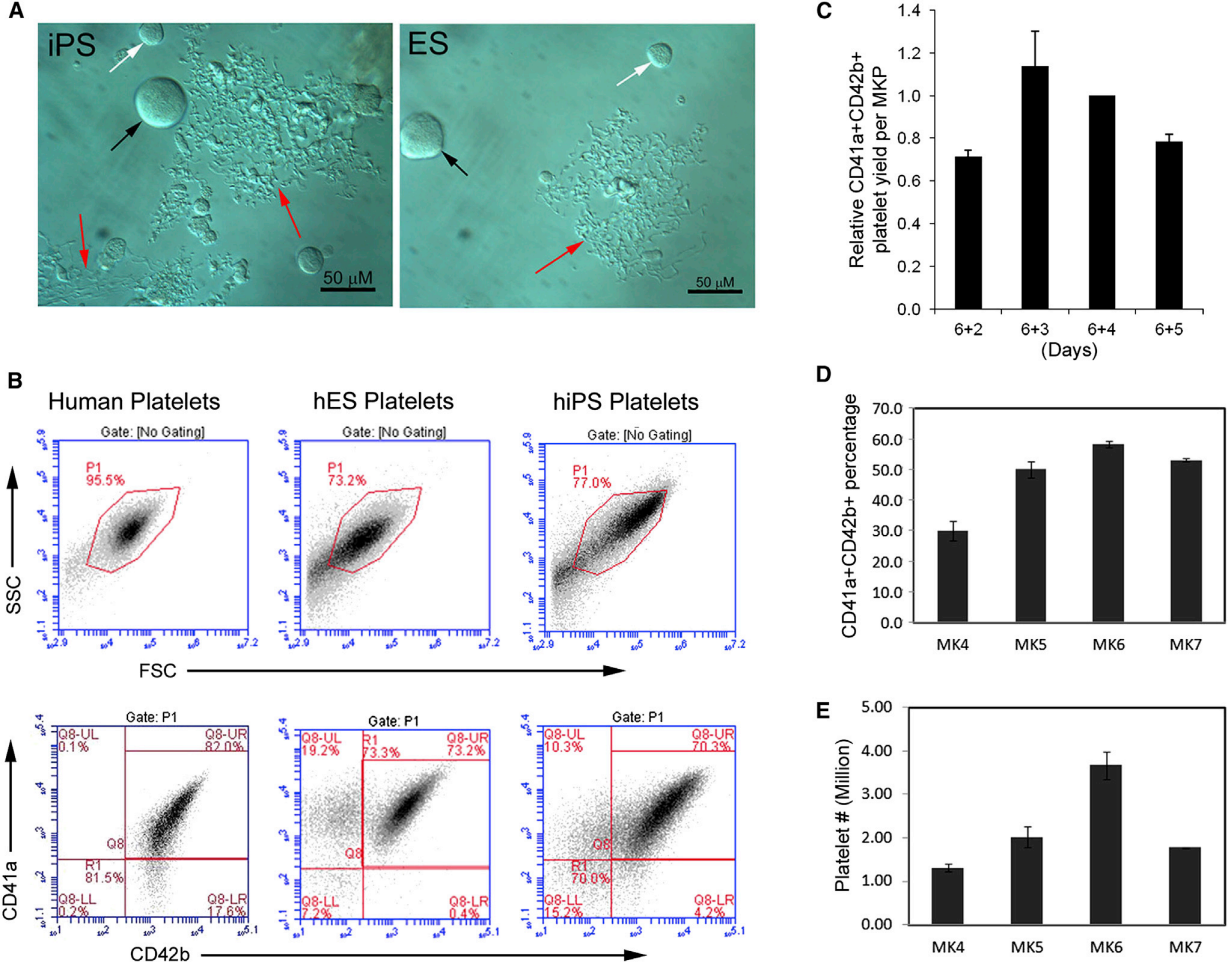
Generation of Platelets from Human iPSC-Derived MKs (A) Morphology of hiPSC- and hESC-derived proplatelets (red arrows), large MKs (black arrows), and small MKs (white arrows). (B) Flow cytometry profile of human circulating platelets, hESC platelets, and iPSC platelets and the percentage of CD41a^+^CD42b^+^ platelets within identical gate (P1). (C) Time-dependent variability of platelet generating capability of MKPs (mean ± SD, n = 3). (D) Time-dependent change of platelet purity during production peak (mean ± SD, n = 3). (E) Time-dependent change of platelet overall yield during production peak (mean ± SD, n = 3).

Although results vary among different cell lines, large MKs (black arrow) and distinctive proplatelet structures (red arrows) were present in 3–4 days of MK cultures (Figure 3A). No difference was observed between MKs derived from iPSC and hESCs in forming proplatelets. To determine if proplatelets in the cultures (Figure 3A) reflect the generation of platelets, we examined CD41a/CD42b expression on iPSC platelets purified by BSA gradient segregation (Robert et al., 2012). Using human blood platelets to establish proper size gating (Figure 3B, left panels), we observed that more than 70% of both iPSC and hESC platelets generated under feeder- and serum-free conditions expressed both CD41a and CD42b, which is similar to human blood platelets (82%). To our knowledge, this is the first time that platelets with such high purity have been generated in vitro, even under conditions with stromal cells and serum. We found that the quality of platelets (CD41a^+^/CD42b^+^ expression) generated from iPSC MKs was inversely correlated with the percentage of CD14^+^ myeloid cells in the starting MK cultures, indicating that a pure MK population is critical for the generation of functional platelets in vitro. The kinetics of platelet production was also monitored, and both the purity and quantity of platelets in culture increased gradually from day 4 and peaked at day 6 (Figures 3C–3E).

Proteases such as MMPs are known to be involved in shedding of CD42b (GPIbα), which is the receptor for vWF and mediates initial platelet reactions to wounds. Loss of CD42b is closely associated with a decline in platelet quality. A broad-spectrum MMP inhibitor, GM6001 (GM), is reported to inhibit CD42b shedding from platelets (Nishikii et al., 2008; Robert et al., 2011). Consistent with our previous study (Lu et al., 2011), the addition of GM in MK culture protected CD42b on platelets from shedding and resulted in an increase of CD41b^+^/CD42b^+^ platelets from 45% to 59% (Figure S3A). However, flow cytometry of GM-treated platelets showed a less homogeneous profile. In a systemic comparative study using several specific MMP inhibitors (unpublished data), we identified an MMP8-specific inhibitor (MMP8-I) with superior shedding protection than GM. Addition of an MMP8-I (20 μM) increased the percentage of CD41a^+^CD42b^+^ platelets from ∼45% to 65%. More importantly, MMP8-I-treated platelets displayed a more homogeneous flow cytometry profile (Figure S3A). The total number of CD41a^+^CD42b^+^ platelets obtained from MMP8-I-treated culture (∼74% increase versus control) was also higher than that from GM-treated cultures (∼46% increase versus control; Figure S3B), However, no significant synergistic effect was observed when combining MMP8-I and GM.

Previous studies have suggested that mild hyperthermia (39°C instead of 37°C) improves MK differentiation/maturation and platelet generation from umbilical cord blood CD34^+^ cells (Pineault et al., 2008; Proulx et al., 2004). However, it is unclear whether mild hyperthermia will be beneficial to iPSC-derived MK differentiation/maturation and platelet production. Using the above-described method, we found that mild hyperthermia (39°C) improved both the purity (percentage of CD41a^+^/CD42b^+^; Figure S4A) and the yield of platelets from iPSC MKs (Figure S3C) during the peak platelet production from day 4 to 7 of MK cultures.

### Ultrastructural and Functional Characterization of iPSC Platelets In Vitro

**Figure 4:**
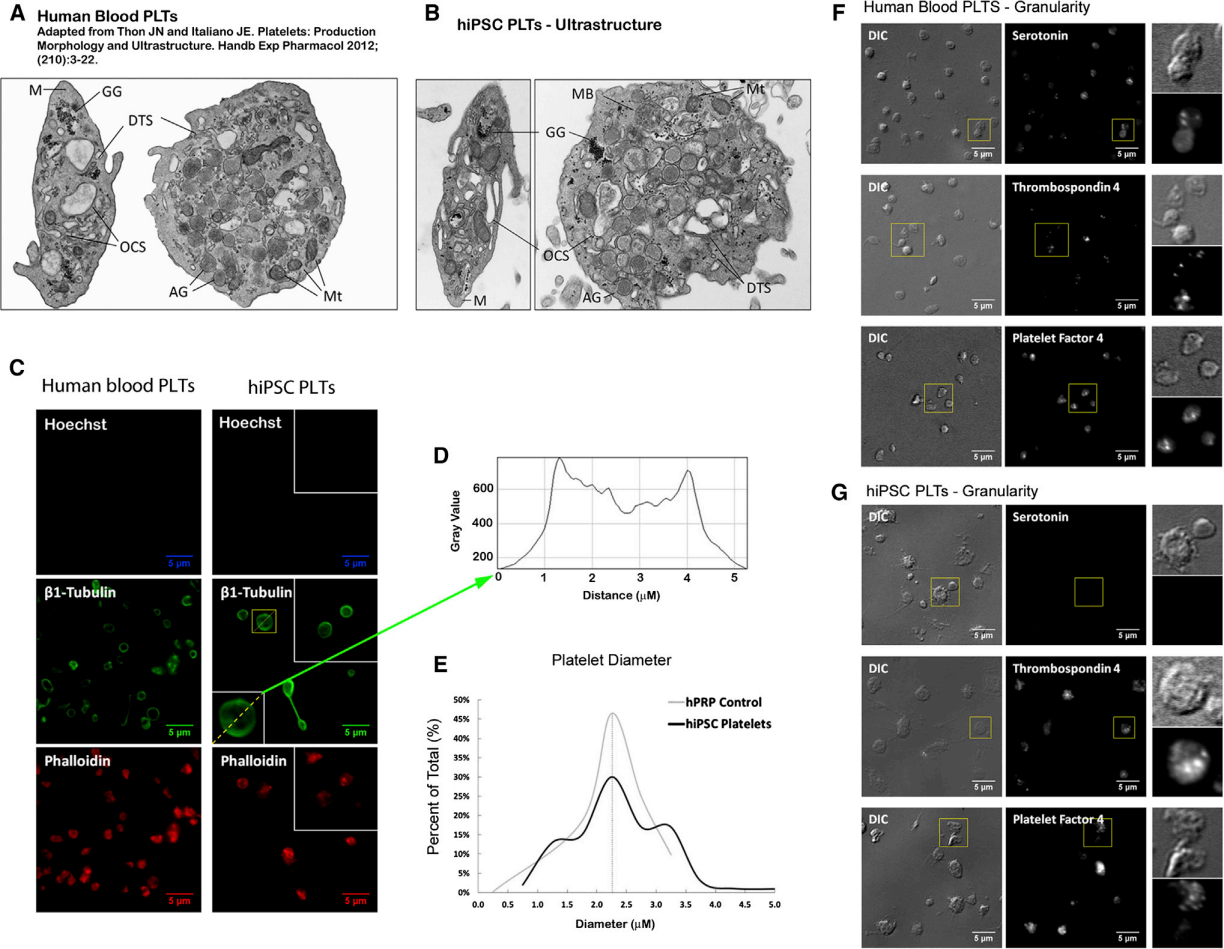
Characterization of iPSC-Derived Platelets In Vitro (A and B) Thin section electron micrographs of (A) human blood and (B) iPSC platelets (AG, α-granules; DTS, dense tubular system; GG, glycogen granules; M, microtubules; MB, multivesicular body; Mt, mitochondria; OCS, open canalicular systems). (C) Immunofluorescence micrograph of human whole blood (left) and iPSC platelets (right). Platelets were probed for Hoechst (nuclear stain, blue), β1-tubulin (microtubule cytoskeleton, green), and phalloidin (filamentous actin, red). (D) Inset (iPSC platelet tubulin) shows representative line function for highlighted platelet. (E) Size distribution of human whole-blood (gray) and iPSC (black) platelets. Platelet diameter was measured in β1-tubulin-labeled cells for more than 100 individual platelets. (F) Granule composition of human whole-blood platelets. (G) Granule composition of iPSC platelets. Platelets were probed for serotonin (dense granule marker), thrombospondin 4, and platelet factor 4 (α-granule markers).

Thin section electron micrography showed that iPSC platelets were ultrastructurally similar to circulating human platelets (Figures 4A and 4B). The iPSC platelets have a discoid shape, display a smooth contour and contain a normal distribution of the open canalicular system as well as α- and dense granules and other organelles, which are indistinguishable from features of human blood platelets (Figures 4A and 4B). Immunofluorescence micrographs showed that iPSC platelets were anucleate but, on average, slightly larger than circulating human blood platelets (2.38 ± 0.85 μm versus 2.27 ± 0.49 μm, n > 100) (Figure 4E). However, size distribution of iPSC platelets was similar to human blood platelets, and these platelets have both normal tubulin cytoskeleton and filamentous actin relative to circulating human platelets (Figure 4E) and strongly express thrombospondin 4 and platelet factor 4 (α-granule markers) (Figures 4F and 4G). In summary, similar to hESC platelets generated with stromal cells (Lu et al., 2011), iPSC platelets displayed all of the ultrastructural and morphological criteria that are characteristic of blood platelets.

**Figure 5:**
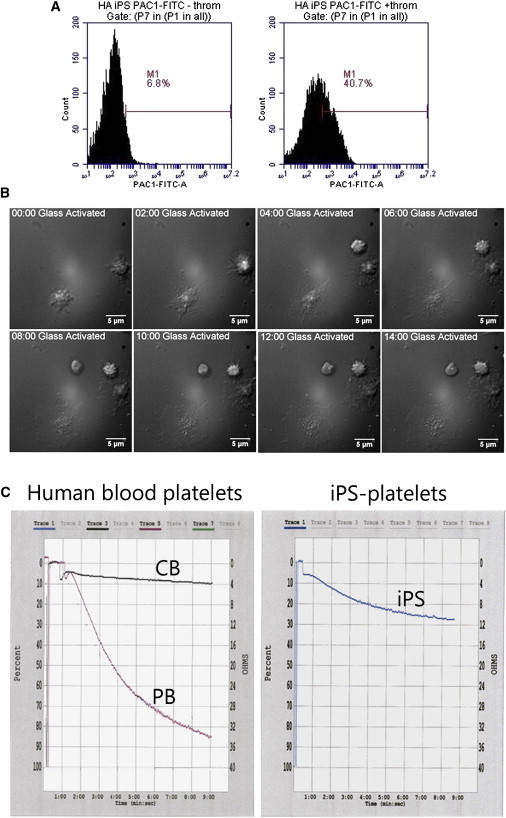
Functional Characterization of iPSC Platelets In Vitro (A) PAC-1 activation of iPSC platelets by thrombin. (B) Time-lapse movie of iPSC platelet spreading upon activation on glass. (C) Aggregation assay of platelets from human peripheral blood (PB), umbilical cord blood (CB), and iPSC platelets stimulated with 1 U of thrombin using Chronolog aggregometer.

To determine whether iPSC platelets generated under this stroma-free condition can be activated, we performed a binding assay using the PAC-1 monoclonal antibody, which only binds to the activated conformation of αIIbβ3 integrin. In response to thrombin treatment, iPSC platelets showed approximately a 6-fold increase in PAC-1 binding as compared to resting controls (Figures 5A and 7C), which is similar to hESC platelets produced with stromal cells but weaker than human blood platelets as reported previously (Lu et al., 2011; Takayama et al., 2008). Live-cell microscopy revealed that iPSC platelets spread on a glass surface and formed lamellipodia and filopodia with membrane ruffling after stimulation; they also spread out and tethered to each other, mimicking the early stage of aggregation, which are characteristics typically observed in blood platelets in response to activation stimulation (Figure 5B; Movie S1).

LTA is the most common method used in clinical and research laboratories to assess platelet function (Panzer and Jilma, 2011). LTA has never been performed with platelets generated from human PSCs in vitro, presumably due to its requirement for a large quantity of fresh platelets. As shown in Figure 5C, human peripheral blood (PB) platelets (2.5 × 10^7^) resuspended in human plasma reached ∼80% aggregation 6 min after exposed to 1 U/ml thrombin. Similar numbers of iPSC platelets responded to 1 U/ml of thrombin in forming aggregates, but the process was slower and aggregation was weaker as compared to PB platelets: only <30% of aggregation was observed 6 min after thrombin stimulation. Human CB platelets similarly showed weak aggregation (∼10%) under the same conditions (Figure 5C), which is consistent with previous observations that CB platelets showed a weaker aggregation compared to PB platelets in the LTA assay (Israels, 2013; Israels and Rand, 2013; Israels et al., 2003). Similar results were observed after exposure of PB and CB platelets and iPSC platelets to 20 μM ADP (Figure S4), the most common agonist used for platelet aggregation studies. However, our results are a significant improvement over in vitro CD34^+^ cell-derived platelets, which showed no aggregation at all by LTA (Robert et al., 2011).

### iPSC Platelets Generated under Completely Serum- and Feeder-free Conditions Are Functional In Vivo

**Figure 6:**
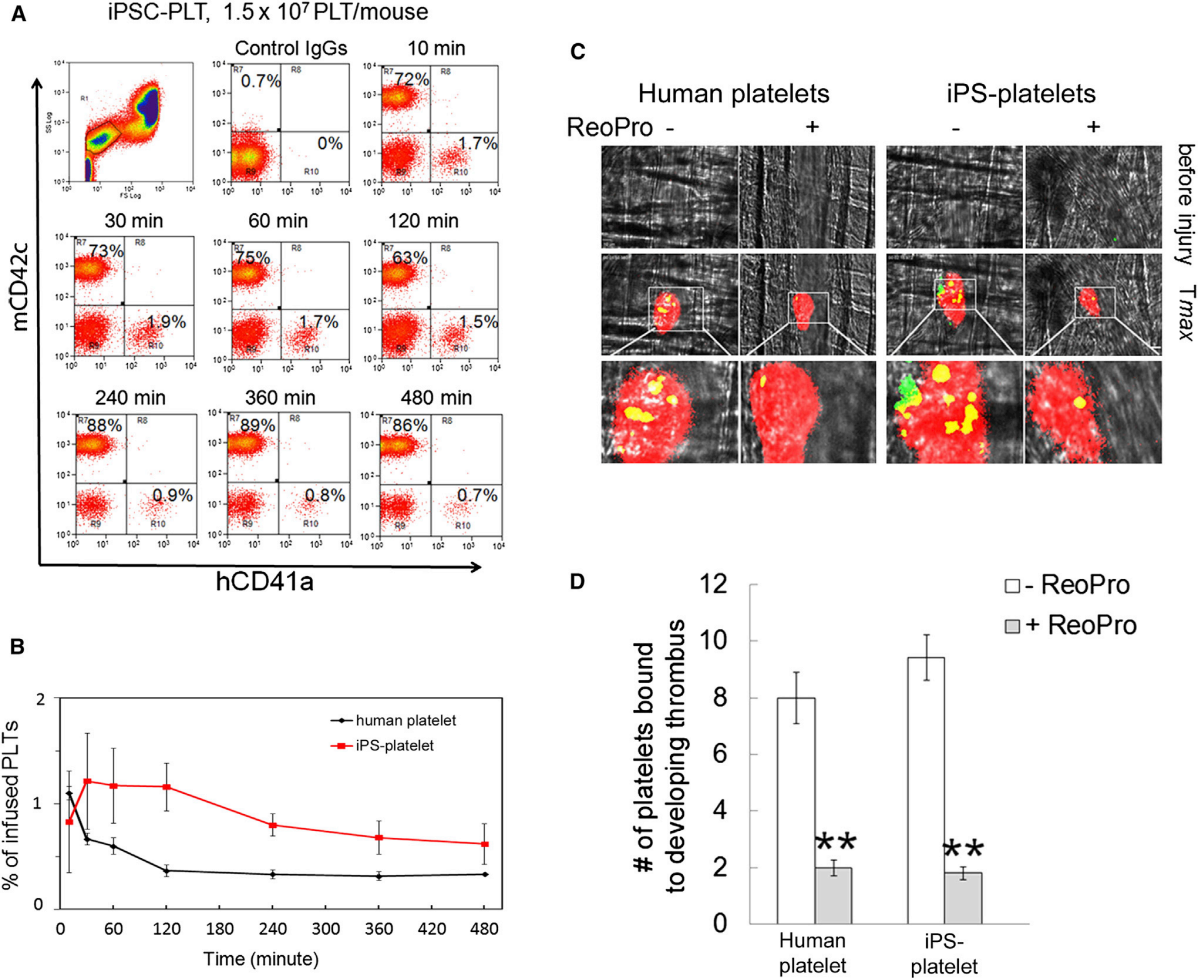
Functional Characterization of iPSC Platelets In Vivo (A) Representative flow cytometry results of iPSC platelet kinetics in macrophage-depleted NOD-SCID mice. (B) Comparative kinetics of human blood platelets and iPSC platelets (mean ± SD, n = 5). (C) In vivo thrombus formation of human blood platelets and iPSC platelets. (D) Quantitative results of in vivo thrombus formation of human whole blood platelets and iPSC platelets (mean ± SEM, n = 6, ^∗∗^p < 0.01); results are from six injuries of three animals.

Previous studies demonstrated that mouse macrophages play a major role in rejecting human platelets (Hu and Yang, 2012). To investigate the kinetics and in vivo functionality of iPSC platelets, nonobese diabetic/severe combined immunodeficiency (NOD/SCID) mice were treated without or with liposome-encapsulated clodronate as described previously (Hu and Yang, 2012), and 1.5 × 10^7^ and 5 × 10^8^ blood human platelets were intravenously infused into these animals. Mouse blood samples were collected at different time points and analyzed by flow cytometry with antibodies specifically against human CD41and CD42 antigens. We observed that human platelets were removed within 10 min from the circulation of liposome-treated control mice (even with the infusion of 5 × 10^8^ human platelets), whereas human platelets circulated for at least 8 hr in macrophage-depleted mice (Figure S5). Therefore, NOD/SCID mice were pretreated with liposome-encapsulated clodronate to deplete macrophages, 1.5 × 10^7^ iPSC platelets/mouse were infused intravenously, and mouse blood samples were analyzed for human platelets at different times. Human iPSC platelets, like human blood platelets, circulated for at least 8 hr in macrophage-depleted NOD/SCID mice with a time to reach maximal accumulation (Tmax) of 0.5–1 hr (Figures 6A and 6B); however, no circulating human blood platelets or iPSC platelets were detected 24 hr after infusion (Figure S6).

We previously reported that hESC platelets, like human blood platelets, incorporated into the developing mouse thrombus at the site of laser-induced arteriolar injury in live mice (Lu et al., 2011). To investigate whether iPSC platelets are functional in vivo, similar experiments were performed in macrophage-depleted NOD/SCID mice. Like human blood platelets and hESC platelets, iPSC platelets incorporated into the growing platelet thrombus with an average number of 9.0 ± 1.8 platelets per thrombus (Figures 6C and 6D; Movie S2), which was indistinguishable from human blood platelets. Infusion of human blood platelets or iPSC platelets did not alter the kinetics of mouse platelet thrombus formation at the site of arteriolar injury (Tmax = 85–105 s). To examine whether the incorporation of iPSC platelets into the developing thrombus is mediated by αIIbβ3 integrin, ReoPro (100 μg), a specific inhibitor of human αIIbβ3 integrin, was infused into the same mouse treated with human platelets or iPSC platelets. Treatment with ReoPro markedly abolished human platelet or iPSC platelet binding to the growing thrombus at the injury sites (Figures 6C and 6D; Movie S3), whereas ReoPro did not affect the number of circulating iPSC platelets or the formation of mouse platelet thrombi (data not shown). These results suggest that iPSC platelets, like human blood platelets and hESC platelets, are functional in vivo.

### Generation of HLA Major-Negative MKs and Platelets from β2M^ko^ iPSCs

**Figure 7:**
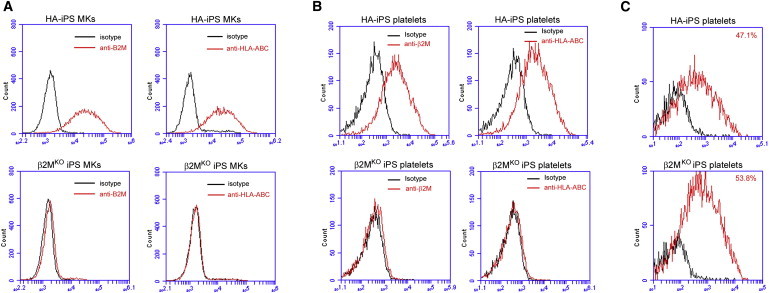
Generation and Characterization of Universal Platelets from Human iPSCs (A) HLA-ABC and β2M expression in wild-type HA-iPSCs and β2M^KO^ iPSC-derived MKs. (B) HLA-ABC and β2M expression in wild-type HA-iPSCs and β2M^KO^ iPSC-derived platelets. (C) Platelet activation measured by PAC-1 binding with (red histogram) or without thrombin (black histogram) in wild-type HA-iPSC platelets and β2M^KO^ iPSC platelets.

MKs and platelets were generated from β2M^ko^ iPSCs, and the expression of both β2M and HLA-ABC was measured with flow cytometry in comparison to those generated from parental HA-iPSCs. The β2M^ko^ iPSCs displayed a normal MK lineage-specific differentiation capability, same as their parental HA-iPSCs. MKs derived from HA-iPSCs, as expected, expressed both β2M and HLA-ABC, while the expression of both antigens in β2M^ko^ iPSC MKs was undetectable (Figure 7A). Similarly, flow cytometry analyses confirmed that platelets generated from β2M^ko^ iPSCs did not express HLA-ABC, whereas platelets derived from parental iPSCs were HLA-ABC positive (Figure 7B). Both HLA-ABC-positive and HLA-ABC-negative platelets possessed similar activation capacity as shown by PAC-1 binding activity after thrombin treatment (47.1% versus 53.8%; Figure 7C).

## Discussion

Here, we describe a completely feeder-free, serum-free, and animal component-free system for the derivation of MKs and platelets from human PSCs, thus rendering the current system amenable to the development of an in vitro current good manufacturing practice-compliant platelet manufacturing protocol. In particular, our use of a hemogenic endothelium intermediate instead of embryoid bodies (EBs) helped avoid inefficiencies and inconsistencies with differentiation of feeder-free PSCs and improved the yield of MK progenitors over other methods. The large-scale generation of pure iPSC platelets allowed us to perform LTA on platelets derived from PSCs. Roughly 4 billion highly purified iPSC platelets were derived using this protocol, and they displayed comparable morphological and functional properties to human blood platelets when tested in a variety of in vitro assays and an intravital/laser-induced thrombosis model in mice.

The formation of EBs and coculture with stroma such as OP9 cells are the two most commonly used methods for differentiation of PSCs into a variety of lineages (Choi et al., 2011; Kennedy et al., 2012; Lu et al., 2007; Takayama et al., 2008). Yet, data suggest that when using EBs, lineage-specific differentiation of feeder-free stem cells is often compromised by significant cell death (Lu et al., 2013) (unpublished data), possibly due to anoikis induced by detachment from the extracellular matrix (ECM). To circumvent the need for EB formation, collagen IV has been used as an ECM to support the differentiation of mouse, nonhuman primate, and human pluripotent stem cell differentiation toward mesoderm lineages (Gerecht-Nir et al., 2003; Nishikawa et al., 1998; Sone et al., 2003). One study by Salvagiotto et al. combined the use of collagen IV with a serum-free medium for early hematopoietic differentiation of feeder-free PSCs and found that the resulting progenitors displayed strong MK lineage potential in MK-CFU assays (Salvagiotto et al., 2011). However, the propensity of these MK progenitors to further differentiate into mature, functional platelets was not examined in any detail (Salvagiotto et al., 2011). We reasoned that if collagen IV could be used in lieu of EBs to support initial differentiation of feeder-free PSCs, its use could thus be incorporated into a full-fledged protocol for the generation of functional platelets. Indeed, we found that 6 days of growth on collagen IV allowed a population of CD31^+^ hemogenic progenitors with hematopoietic potential to emerge from the starting iPSCs. These CD31^+^ cells could then be efficiently differentiated into MK progenitors, consistently generating ∼16 MK progenitors from every 1 iPSC. This is a significant improvement over previously reported methods and allowed us to produce billions of MK progenitors in conventional culture vessels.

Subsequent differentiation of stem cell-derived MK progenitors into mature MKs and ultimately platelets has been notoriously difficult. Coculture with OP9 or C3H10T1/2 stroma cells has been found to greatly facilitate the process (Lu et al., 2011; Takayama et al., 2008), but it is still far from being efficient. We found that optimizing the combination of medium and cytokines at each unique phase of MK lineage-specific differentiation and maturation together with the use of ultralow-attachment plates can overcome the necessity for stroma/MK coculture to generate high-quality, functional platelets. Indeed, we produced more pure platelets under this feeder-free system than those of previous studies using feeder cells (Lu et al., 2011; Takayama et al., 2008), suggesting that under the right circumstances, feeder cells are entirely dispensable for platelet formation in vitro. Our results also highlight the importance of media and matrix optimization for future investigation.

Despite these improvements, the current output of approximately six platelets per MKP is still quite low compared to platelet output in adult BM, where a mature MK is capable of producing 2,000–10,000 platelets (Kaufman et al., 1965; Long, 1998). This may be due to the inadequacy of static in vitro culture conditions and/or the lack of a pro-MK niche mimicking the BM sinusoid (Junt et al., 2007). Using microfluidic chips, we have recently demonstrated that approximately 30 platelets per iPSC MK were generated in vitro under sheer force (Thon et al., 2014), a 5-fold increase in efficiency compared to the static condition used in current study. Although this progress is significant, the efficiency is still very low compared to >2,000 platelets/MK in BM. Currently, it remains the biggest challenge for reaching a clinically relevant scale of ex vivo platelets. One strategy for getting around this problem involves the generation of immortalized megakaryocyte progenitor cell lines (imMKCLs) from hematopoietic progenitors with virally transduced genes. These imMKCLs can be expanded and are capable of producing platelets upon inhibition of c-*myc*, *bmi*1, and *bcl*-xL expression (Nakamura et al., 2014). Although methods involving viral transduction may not be considered appropriate for clinical-grade manufacturing, this new study offers insight into mechanisms behind megakaryopoiesis that may help develop clinically compliant protocols. We found that the efficiency of MK progenitor generation and subsequent platelet production can be enhanced by transient inhibition of c-*myc* expression. Our data indicate that c-*myc* inhibition using the small molecule iBET151 leads to increased expression of the erythroid/MK lineage-specific transcription factor GATA-1 and a concomitant decrease in the production of CD14^+^ myeloid cells, both of which likely enhance MK differentiation and lead to increased platelet production. These observations are in line with previous reports showing that timely alterations in c-*myc* levels achieved by inducible c-*myc* systems (Takayama et al., 2010; Thompson et al., 1996) or completely knocking out c-*myc* expression (Guo et al., 2009) can affect erythroid/MK lineage commitment, megakaryopoiesis, polyploidy, and platelet output. As iBET151 is slightly cytotoxic, more work will need to be done to optimize the concentration and timing of its use for maximal benefit.

Finally, we provided proof of concept that our feeder-free, serum-free, and animal-component free system can be used to generate HLA-ABC^neg^, universal platelets by using TALEN-mediated targeting disruption of the β2-microglobin gene in iPSCs. Knocking out β2M expression eliminates HLA class I cell-surface expression (D’Urso et al., 1991), which is thought to be a major cause for platelet refractoriness (Hod and Schwartz, 2008). The potential clinical importance of β2M/HLA class I disruption is further supported by a previous study showing that MKs generated from β2M knockdown CD34^+^ cells could produce HLA-negative platelets upon injection into immunocompromised mice. Importantly, these HLA-negative platelets avoided immune detection induced by injection of specific anti-HLA antibodies (Gras et al., 2013). With future efforts aimed at improving the efficiency of in vitro platelet generation from MKs, the application of β2M null iPSCs could one day provide a replenishable supply of universal platelets for alloreactive patients.

## Experimental Procedures

### Reagents

STEMspan-ACF and STEMdiff-APEL Media, STEMspan Megakaryocyte Expansion Supplement, mTeSR1, Dispase, and CryoStor CS10 were purchased from STEMCELL Technologies. BMP4 was from HumanZyme. All other cytokines were obtained from PeproTech. Y-27632 was purchased from Stemgent. Human Collagen IV was from Advanced BioMatrix. Matrigel and antibodies for flow cytometry were obtained from BD Biosciences. I-BET 151(GSK1210151A) was purchased from ChemieTek. MMP-8 Inhibitor I (CAS 236403-25-1) was purchased from Millipore. StemPro Accutase was from Life Technologies. Heparin was purchased from Sigma.

### Human Pluripotent Stem Cell Cultures

The human iPSC line HA1-iPS was reprogrammed by mRNA (Warren et al., 2012) and obtained from Allele Biotechnology. The human iPSC lines 19-9-11T and 6-9-9T (WiCell) were reprogrammed with episomal vectors. The human iPSC line RHO8 was generated with retroviral vectors using fibroblasts derived from a RH- O blood type donor. The human HDF-iPSC XA line (XA-iPS) was generated from human dermal foreskin fibroblasts using 6F Reprogramming Premix (Allele Biotechnology). The hESC lines MA09 and NED07 were derived using single blastomeres (Klimanskaya et al., 2007). All human pluripotent stem cells were cultured on a Matrigel-coated surface with mTeSR1 medium. Confluent pluripotent stem cells were dissociated either with dispase (1 U/ml, STEMCELL Technologies) or cell dissociation buffer (CDB, Life Technologies). All pluripotent stem cells used in this study have normal karyotypes.

### Generation of β2M^KO^ iPSCs

Derivation of β2M^KO^ iPS was performed by Cellectis Bioresearch using TALEN technology to disrupt the β2M gene. Briefly, HA-iPSCs were transfected with a hsβ2M TALEN targeting exon 2, β2M^KO^ single cells were sorted by FACS, and the TALEN-mediated deletion in established β2M-negative iPSC clones was validated by deep sequencing. Lack of β2M expression was confirmed by flow cytometry. The knockout engineering of HA-iPSCs was performed under feeder-free and xeno-free culture condition.

### MK-and Platelet-Specific Differentiation of Pluripotent Stem Cells

Feeder-free iPSCs were dissociated with CDB and resuspended in fresh mTeSR1 medium containing 10μM of Y27632. Cells were seeded on human collagen IV-coated plates (5 μg/cm^2^) and incubated at 37°C in 5% CO_2_, 20% O_2_ for 24 hr. Media were then changed to STEMspan-ACF + BMP4, vascular endothelial growth factor, and basic fibroblast growth factor (50 ng/ml each), and cells were grown for 4 days under hypoxic conditions (5% CO_2_, 5% O_2_) followed by 2 additional days at 5% CO_2_, 20% O_2_. On day 6, cells were analyzed for cell-surface markers CD31, CD34, CD43, CD144 (VE cadherin), CD105 (Endoglin), CD184 (CXCR4), and CD309 (KDR) by flow cytometry (Accuri C6).

To promote early MKP production, cells were cultured in MK-Specific Progenitor Expansion medium (EXP-M) containing STEMdiff APEL Medium + thrombopoietin (TPO) (25 ng/ml), stem cell factor (SCF) (25 ng/ml), Flt-3 ligand (25 ng/ml), interleukin-3 (IL-3) (10 ng/ml), IL-6 (10 ng/ml), and heparin (5 U/ml) for up to 7 days. Nonadherent MKPs were harvested for 4–5 consecutive days in EXP-M and cryopreserved in CryoStor medium. Samples of MKPs were checked for CD41a, CD42b, CD31, CD34, CD43, CD13, CD235a, and CD14 expression by flow cytometry.

To induce MK maturation and platelet formation, MKPs were cultured in MK maturation medium (MK-M) containing STEMSpan-ACF + TPO, SCF, IL-6 and IL-9 and heparin (5 U/ml) in ultralow attachment plates (Corning). Five micromolar Y-27632 was added for the first 3 days of culture, and cells were incubated in 7% CO2 at 39°C. Cell densities were monitored daily and fresh medium was added to maintain 10^6^ cells/ml for the first 4 days. The maturation of MKs from MKPs was monitored by analyzing CD41a, CD42b, and CD235a expression. Once proplatelet morphology (Figure 3A) was observed, platelets were collected for 3–5 consecutive days and analyzed for CD41a/CD42b expression.

To extract platelets, large MKs were removed first using low-speed centrifugation (50 × *g* for 10 min), and proplatelets and small MKs were removed by further 10 min centrifugation at 300 × *g*. iPSC platelets were collected using higher *g* force (1,000–2,500 × *g*) in the presence of PGE1 (1 μM). The collected iPSC platelet pellets were then subjected to further purification using a BSA gradient centrifugation as described previously (Robert et al., 2012).

### Human Blood Platelet Preparation and In Vitro Microscopic Characterizations

Human blood was obtained by venipuncture from healthy volunteers, as previously described (Thon et al., 2012). Collections were performed in accordance with ethics regulation with IRB approval, and informed consent was provided according to the Declaration of Helsinki. For electron microscopy, both human blood and iPSC platelets were fixed and ultrathin sections were stained and examined with a Tecnai G2 Spirit BioTwin electron microscope as reported previously (Lu et al., 2011).

Differential interference contrast analysis was performed as previously reported (Lu et al., 2011). For microtubule components, samples were stained with an anti-β1-tubulin antibody (Genemed Synthesis); for actin components, samples were incubated with Alexa Fluor 568-conjugated phalloidin (Invitrogen). To confirm cells were anucleate, samples were incubated with Hoechst (Invitrogen). For granule localization, samples were incubated with antibodies against serotonin (EMD Millipore), thrombospondin 4 (Neomarkers), or platelet factor 4 (Peptrotech) and treated with secondary antibodies, all conjugated to Alexa 488 (Invitrogen).

For contact-activated spreading time lapse, iPSC platelets were pipetted into chambers formed by mounting a glass coverslip onto a 10 mm Petri dish with a 1 cm hole. Platelets were permitted to contact glass by gravity sedimentation, and spreading was captured at 5 s intervals over a 5 min period.

For size determination, platelets were individually thresholded from β1-tubulin-labeled samples, and high-content diameter measurements were performed in ImageJ using the linescan and measurement functions. Analysis was confirmed by manual inspection of all samples, and improperly thresholded cells were excluded from the analysis. More than 100 cells were counted for each condition.

### Platelet Kinetics in Live Animals

Macrophages were depleted in NOD/SCID mice (6–7 weeks old, male) by intravenous injection of liposome-encapsulated clodronate as described previously (Hu and Yang, 2012). At day 3, human blood platelets and iPSC platelets were intravenously infused into macrophage-depleted mice. Blood (30 μl) was collected at different time points (10, 30, 60, 120, 240, 360, and 480 min and 24 hr) and analyzed by flow cytometry using allophycocyanin (APC)-conjugated anti-human CD41 and Dylight 488-conjugated anti-mouse CD42c antibodies. The University of Illinois Institutional Animal Care and Use Committee approved all animal care and experimental procedures.

### Real-Time Fluorescence Intravital Microscopy

Intravital microscopy of cremaster muscle arterioles was performed as previously described (Lu et al., 2011). NOD/SCID mice were depleted for macrophages, and the cremaster muscle arteriolar wall was injured by a micropoint laser. The developing mouse platelet thrombus was visualized by infusion of Dylight 649-conjugated anti-mouse CD42c antibodies (Emfret Analytics, 0.05 μg/g body weight), and calcein AM-labeled human platelets and iPSC platelets (3∼10^6^/mouse) were then infused with or without ReoPro (100 μg) into mice. Fluorescence and bright-field images were recorded and data were collected for 5 min following vessel wall injury and analyzed using Slidebook v5.5 (Intelligent Imaging Innovations). The University of Illinois Institutional Animal Care and Use Committee approved all animal care and experimental procedures.

### Light Transmission Aggregometry and PAC-1 Activation Assay

Human iPSC-derived platelets or human peripheral and cord blood platelets were counted on a Sysmex Hematoanalyzer XE-2100D, spun down (15,000 × *g*, 15 min), and resuspended in normal human plasma at the same concentration for light transmission aggregometry. A total of 25 million platelets suspended in plasma (270 μl) were placed in Chronolog cuvettes with stir bars at 1,000 rpm. Normal human plasma was used as a blank. The baseline was set on Aggrolink software, and agonist (30 μl) was added to a final concentration of 20 μM for ADP or 1 U/mL for thrombin (final volume = 300 μl). Data were collected for 10 min, and the aggregation percentage was calculated with Aggrolink software. PAC-1 activation assay was performed as previously reported (Lu et al., 2011).

### Expression of β2M and HLA-ABC on HA-iPSCs and β2M^ko^ iPSC-Derived MKs and Platelets

Wild-type HA-iPSCs and β2M^ko^ iPSCs were differentiated into MKs and platelets using the method described above. Mature MKs from both lines were stained with fluorescein isothiocyanate (FITC) anti-human β2M antibody (BioLegend, Clone 2M2) and FITC anti-human HLA-A,B,C (BioLegend, clone W6/32). FITC-isotype immunoglobulin Gs were used as control. Platelets generated from HA-iPSCs and β2M^ko^ iPSCs were stained with phycoerythrin anti-human CD42b (BD) and APC anti-human CD41a (BD) together with either FITC anti-human β2M or FITC anti-human HLA-A,B,C antibodies. PAC1 activation of platelets was performed as described previously.

### Statistical Analysis

Data were statistically analyzed by Student’s t test for comparison of two groups. Differences were considered significant at p < 0.05. “n” stands for independent experiments unless otherwise described.

## Author Contributions

Q.F., S.J.L., and R.L. conceived and designed the experiments. Q.F., N.S., J.N.T., H.H., A.T., K.R.M., K.K., J.B., F.L., C.L., J.I., and J.C. performed the experiments; J.W. and K.S.K. contributed materials; Q.F., S.J.L., E.A.K., and R.L. wrote the paper.

## Supplemental Information

Document S1. Figures S1–S6 and Table S1

Movie S1. Activation of iPSC Platelets In Vitro Live-cell video microscopy of activating platelets was performed using previously described methods (Lu et al., 2011). Images of spreading platelets were captured every 5 seconds for 10 minutes with Metamorph software. Structural changes that occur following activation of iPSC platelets were observed using differential interference contrast optics in a light microscope. iPSC platelets were found to spread and form broad, flat lamellipodia and finger-like filopodia. Ruffling activity was observed along the cell edges, and some of them were tethered together.

Movie S2. Incorporation of iPSC Platelets in Developing Thrombus without Inhibitor iPSC platelets incorporate into the developing mouse platelet thrombus at the site of laser-induced arteriolar injury in living mice. Dylight 649-labeled anti-mouse CD42 (0.05 μg/g body weight) was infused to monitor a mouse platelet thrombus. Calcein AM-labeled iPSC platelets (50–100 μl [3 × 106 platelets]) were infused through a femoral artery cannulus immediately after laser-induced arteriolar wall injury. Mouse platelets (red) accumulated as fast as 5–20 seconds after vessel injury. Circulating iPSC platelets and iPSC platelets incorporated into the developing mouse platelet thrombus are shown in green and yellow, respectively.

Movie S3. Incorporation of iPSC Platelets in Developing Thrombus with the αIIbβ3-Specific Inhibitor ReoPro iPSC platelets incorporate into the developing mouse platelet thrombus in an αIIbβ3-dependent manner at the site of laser-induced arteriolar injury in living mice. Dylight 649-labeled anti-mouse CD42 (0.05 μg/g body weight) was infused to monitor a mouse platelet thrombus. Calcein AM-labeled iPSC platelets (50–100 μl [3 × 106 platelets]) were pretreated with the αIIbβ- specific inhibitor ReoPro (100 μg/mouse) and infused through a femoral artery cannulus immediately after laser-induced arteriolar wall injury. Mouse platelets (red) accumulated as fast as 5–20 seconds after vessel injury. Pretreatment with ReoPro reduced the number of human iPSC platelets within the growing mouse platelet thrombus. Circulating iPSC platelets and iPSC platelets incorporated into the developing mouse platelet thrombus are shown in green and yellow, respectively.

Document S2. Article plus Supplemental Information
