# Supplementary material for: Scalable Generation of Universal Platelets from Human Induced Pluripotent Stem Cells
Source: Stem Cell Reports. 2014 Oct 16;3(5):817–31. doi: 10.1016/j.stemcr.2014.09.010 (PMC4235139; doi:10.1016/j.stemcr.2014.09.010)
Supplement: Document S1. Figures S1–S6 and Table S1 [file mmc1.pdf]

Stem Cell Reports, Volume *3*
Supplemental Information

Scalable Generation of Universal Platelets from Human Induced Pluripotent Stem Cells

Qiang Feng, Namrata Shabrani, Jonathan N. Thon, Hongguang Huo, Austin Thiel, Kellie R. Machlus, Kyungho Kim, Julie Brooks, Feng Li, Chenmei Luo, Erin A. Kimbrel, Jiwu Wang, Kwang-Soo Kim, Joseph Italiano, Jaehyung Cho, Shi-Jiang Lu, and Robert Lanza

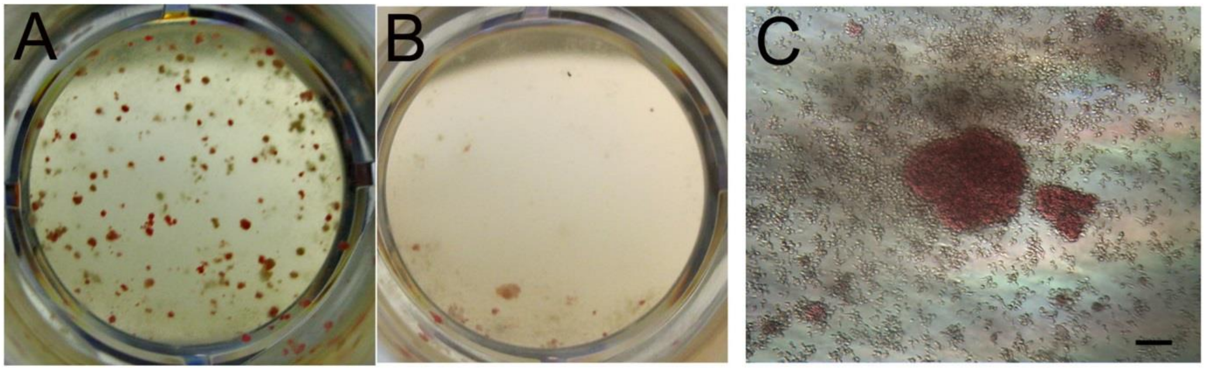

Supplement Figure 1. **CFU development from CD31+ hemogenic endothelium-like cells**. Representative CFU-forming culture wells (24-well) of Day 6 (A) CD31$^+$ hemogenic endothelium-like cells, (B) CD31$^-$ cells and (C) high magnification image of representative CFUs (scale bar = 100 μM)

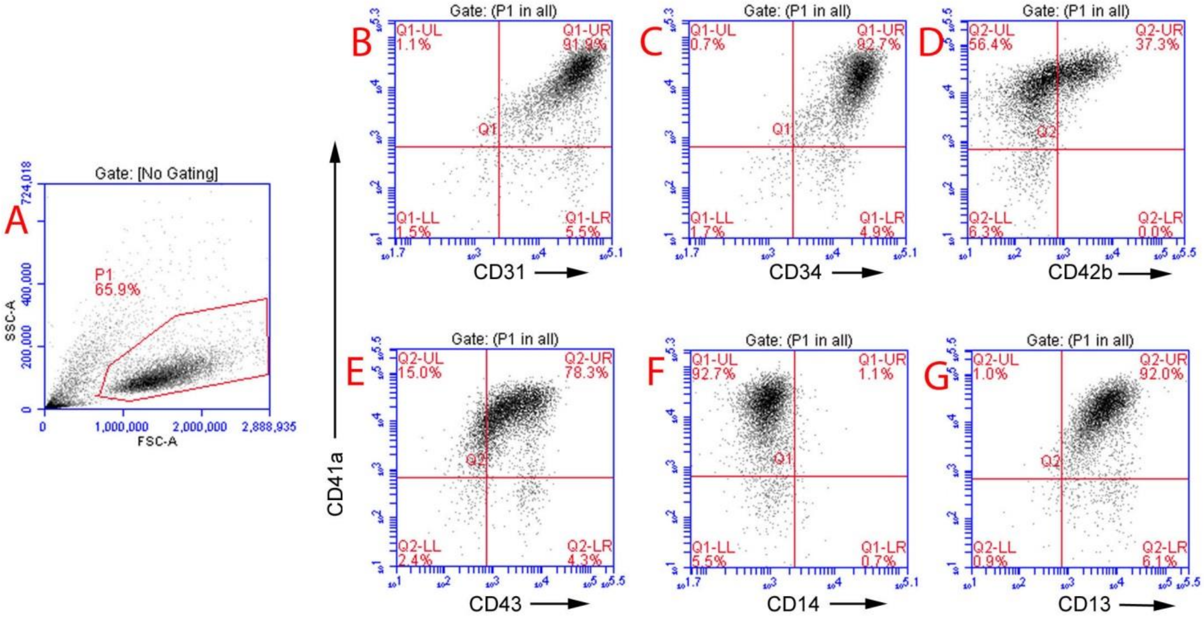

Supplement Figure 2. **Surface marker characterization of iPS-megakaryocyte progenitors (MKP).** Representative results of cell surface marker analysis of iPS-derived MKPs. **A**: SSC and FSC of MKPs (P1); **B**: CD31; **C**: CD34; **D**: CD42b; **E**: CD43; **F**: CD14; **G**: CD13. (All samples were also co-stained for CD41a)

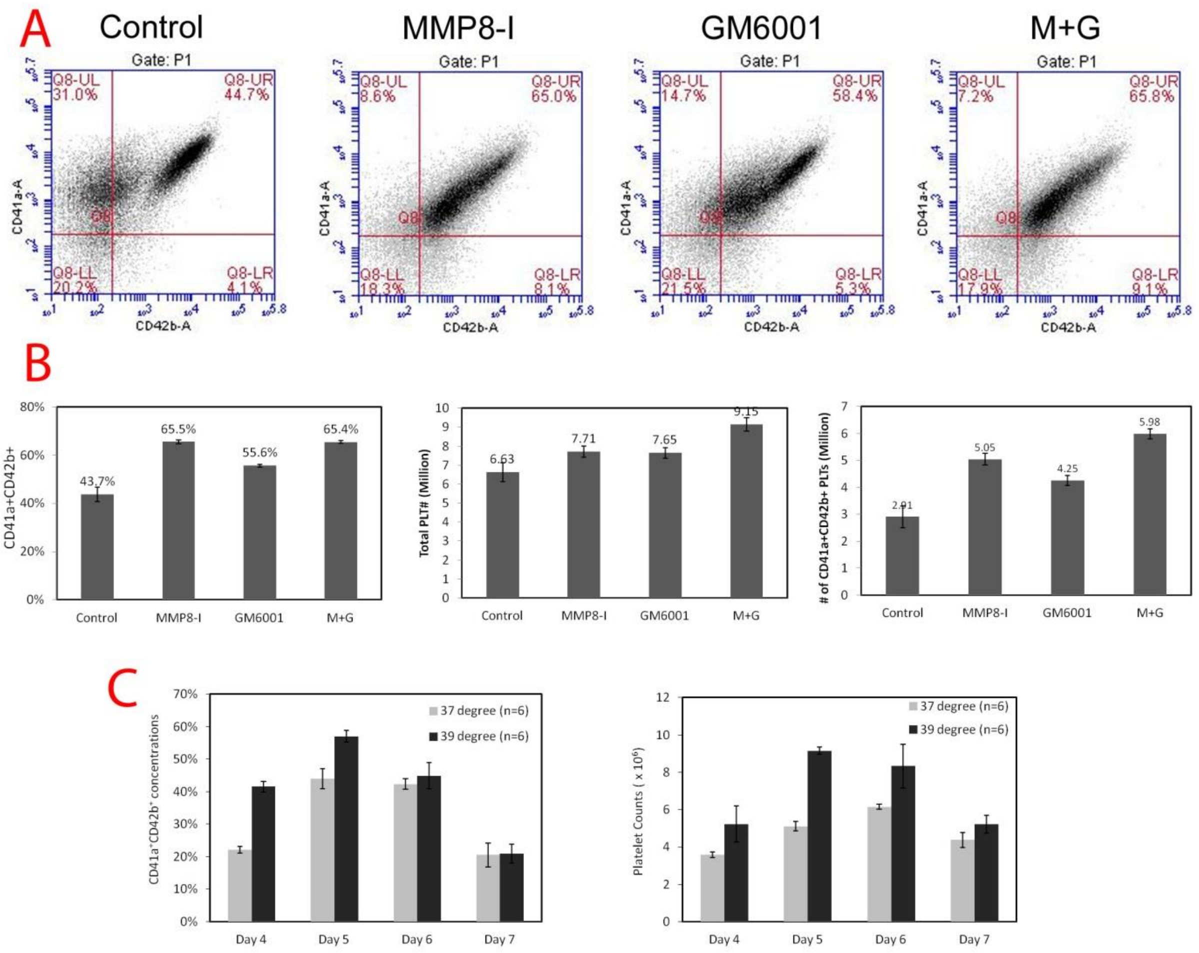

Supplement Figure 3. Effects of MMP inhibitors and mild hyperthermia on platelet generation. A: Representative FACS profiles showing effect of control, GM6001, MMP8-I and GM6001/MMP8-I treatment on iPS-platelet purity. B: Effect of GM6001, MMP8-I and GM6001/MMP8-I treatment on platelet purity and yield (MK4-7 indicate days of MK culture, Mean ± SD, n=3); C: Effect of mild hyperthermia condition (39°C) on platelet purity and yield (MK4-7 indicate days of MK culture, Mean ± SD, n=6).

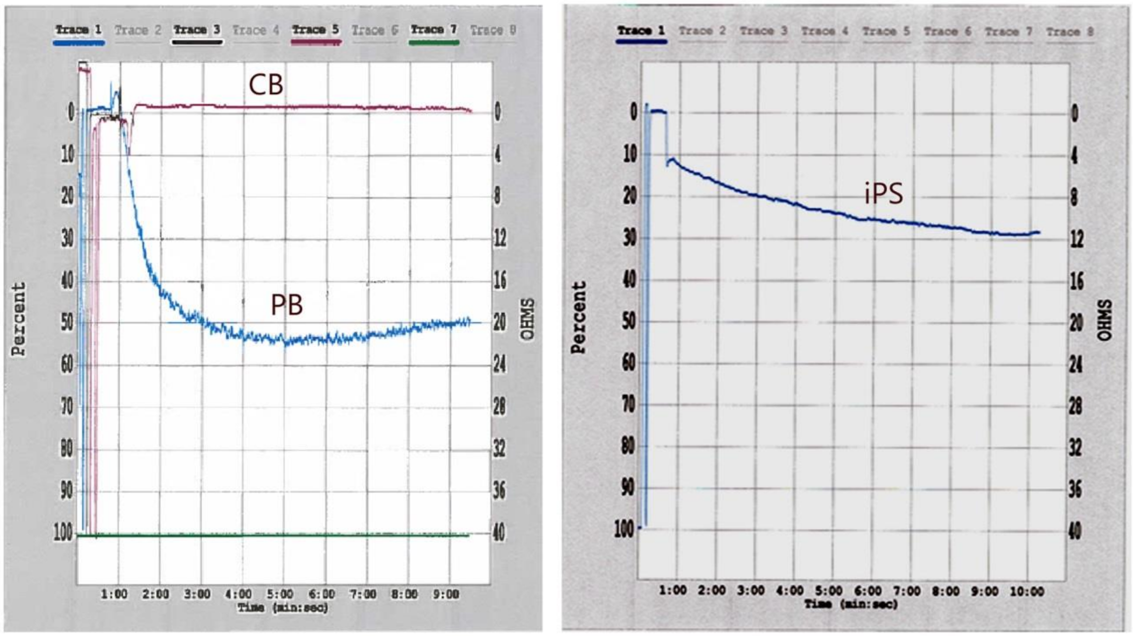

20 millions of platelets from peripheral blood (PB), cord blood (CB) and iPS cells were stimulated with 20 uM of ADP, aggregations were monitored on a Light Transmission Aggregometor (LTA)

Supplement Figure 4. Light transmission aggregation assay of PB, CB and iPS-derived platelets. Aggregation assay of platelets from human peripheral blood (PB), umbilical cord blood (CB) and iPS-platelets stimulated with 20 uM ADP using Chronolog aggregometer

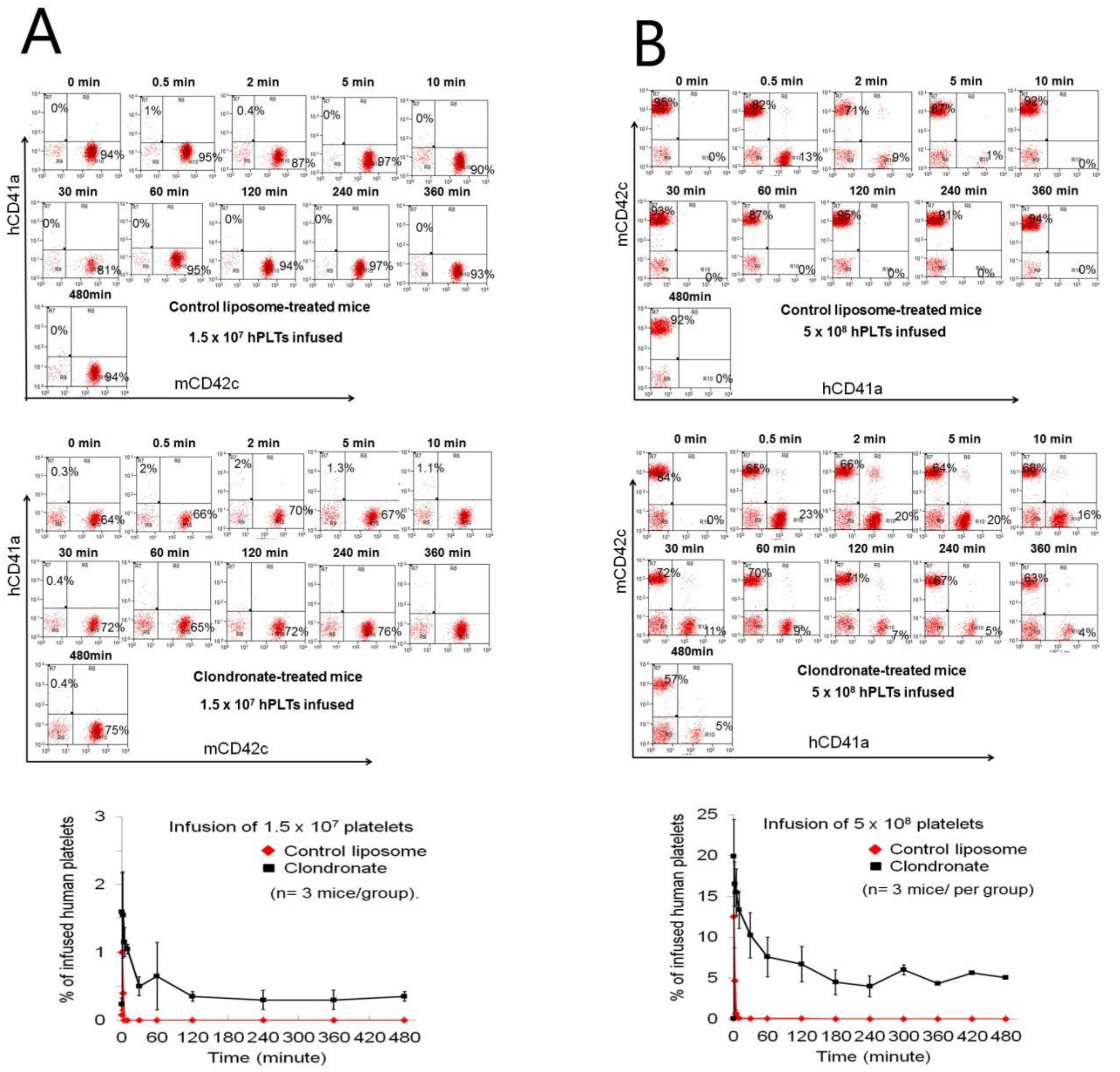

Supplement Figure 5. Kinetics of human blood platelet circulation in mouse. Representative FACS results of human platelet kinetics in control and macrophage-depleted NOD-SCID mice; A: 1.5 x 10^7 human platelets; B: 5 x 10^8 human platelets. Mean ± SD, n=3.

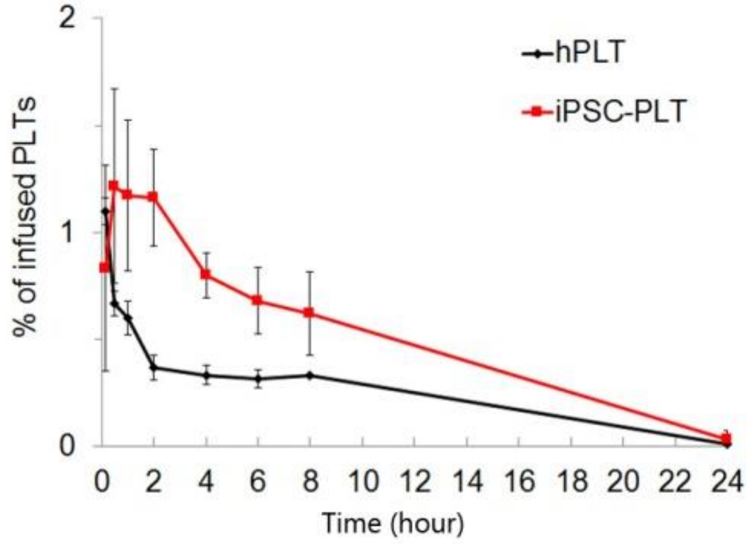

Supplement Figure 6. Comparative kinetics of human blood platelets and iPS-platelets in macrophage-depleted NOD-SCID mice for 24 hours after initial infusion. Mean ± SD, n=5.

Supplement Table. MK progenitor yield per iPS cell. (Mean ± SD, n=5).

	**Total MKP collected (Million)**	**Starting iPSC Number (million)**	**MKPs generated per iPSC**
Exp 1	497.6	27	18.4
Exp 2	254	18	14.1
Exp 3	430	27	15.9
Exp 4	462	27	17.1
Exp 5	414	27	15.3
	Total = 2058	Total = 126	16.16 ± 1.655 (Mean ± SD, n=5)

Supplement Video 1. **Activation of iPS-platelets in vitro**. Live cell video microscopy of activating platelets was performed using previously described methods[5] (Lu et al, Cell Res. 2011;21(3):530-545). Images of spreading platelets were captured every 5 seconds for 10 minutes with Metamorph software. Structural changes that occur following activation of iPS-platelets were observed using differential interference contrast optics in a light microscope. iPSC-platelets were found to spread, form broad flat lamellipodia and finger-like filopodia. Ruffling activity was observed along the cell edges, and some of them were tethered together.

Supplement Videos 2 and 3. Incorporation of iPS-platelets in developing thrombus. iPS-platelets incorporate into the developing mouse platelet thrombus in an αIIbβ3-dependent manner, at the site of laser-induced arteriolar injury in living mice. Dylight 649-labeled anti-mouse CD42 (0.05 µg/g body weight) was infused to monitor a mouse platelet thrombus. Calcein AM-labeled iPS-platelets, 50-100 µl (3 x 10^6 platelets), were pretreated without (Video 2) or with (Video 3) ReoPro (100 µg/mouse) and infused through a femoral artery cannulus immediately after laser-induced arteriolar wall injury. Mouse platelets (red) accumulated as fast as 5-20 seconds after vessel injury. Pretreatment with ReoPro reduced the number of human iPS-platelets within the growing mouse platelet thrombus. Circulating iPS-platelets and iPS-platelets incorporated into the developing mouse platelet thrombus are shown in green and yellow, respectively.
